# The Effect of a Combined Hydrogen Peroxide-MlrA Treatment on the Phytoplankton Community and Microcystin Concentrations in a Mesocosm Experiment in Lake Ludoš

**DOI:** 10.3390/toxins11120725

**Published:** 2019-12-11

**Authors:** Dariusz Dziga, Nada Tokodi, Damjana Drobac, Mikołaj Kokociński, Adam Antosiak, Jakub Puchalski, Wojciech Strzałka, Mariusz Madej, Zorica Svirčev, Jussi Meriluoto

**Affiliations:** 1 Department of Microbiology, Faculty of Biochemistry, Biophysics and Biotechnology, Jagiellonian University, Gronostajowa 7, 30387 Krakow, Poland; adam.antosiak@student.uj.edu.pl (A.A.); puchal1610@gmail.com (J.P.); mariusz.madej@doctoral.uj.edu.pl (M.M.); 2 Department of Biology and Ecology, Faculty of Sciences, University of Novi Sad, Trg Dositeja Obradovića 2, 21000 Novi Sad, Serbia; nada.tokodi@dbe.uns.ac.rs (N.T.); biometatandem@gmail.com (D.D.); svircevzorica@gmail.com (Z.S.); jussi.meriluoto@abo.fi (J.M.); 3 Department of Hydrobiology, Faculty of Biology, Adam Mickiewicz University, Uniwersytetu Poznańskiego 6, 61-614 Poznań, Poland; kok@amu.edu.pl; 4 Department of Plant Biotechnology, Faculty of Biochemistry, Biophysics and Biotechnology, Jagiellonian University, Gronostajowa 7, 30387 Krakow, Poland; strzalkawojciech@gmail.com; 5 Biochemistry, Faculty of Science and Engineering, Åbo Akademi University, Tykistökatu 6 A, 20520 Turku, Finland

**Keywords:** microcystin, biodegradation, hydrogen peroxide, treatment, cyanobacteria, bloom

## Abstract

Harmful cyanobacteria and their toxic metabolites constitute a big challenge for the production of safe drinking water. Microcystins (MC), chemically stable hepatotoxic heptapeptides, have often been involved in cyanobacterial poisoning incidents. A desirable solution for cyanobacterial management in lakes and ponds would eliminate both excess cyanobacteria and the MC that they potentially produce and release upon lysis. Hydrogen peroxide (H_2_O_2_) has recently been advocated as an efficient means of lysing cyanobacteria in lakes and ponds, however H_2_O_2_ (at least when used at typical concentrations) cannot degrade MC in environmental waters. Therefore, mesocosm experiments combining the cyanobacteria-lysing effect of H_2_O_2_ and the MC-degrading capacity of the enzyme MlrA were set up in the highly eutrophic Lake Ludoš (Serbia). The H_2_O_2_ treatment decreased the abundance of the dominant cyanobacterial taxa *Limnothrix* sp., *Aphanizomenon flos-aquae*, and *Planktothrix agardhii*. The intracellular concentration of MC was reduced/eliminated by H_2_O_2_, yet the reduction of the extracellular MC could only be accomplished by supplementation with MlrA. However, as H_2_O_2_ was found to induce the expression of *mcyB* and *mcyE* genes, which are involved in MC biosynthesis, the use of H_2_O_2_ as a safe cyanobacteriocide still requires further investigation. In conclusion, the experiments showed that the combined use of H_2_O_2_ and MlrA is promising in the elimination of both excess cyanobacteria and their MC in environmental waters.

## 1. Introduction

Treatment with hydrogen peroxide (H_2_O_2_) is one of the contemporary proposals for effective control of cyanobacterial bloom formation. However, there are contradictory opinions on how this kind of treatment influences the actual concentration of cyanotoxins such as microcystins (MC). Successful elimination of MC requires both H_2_O_2_-induced lysis of cyanobacteria and degradation of the released MC. The physiological response of cyanobacteria to H_2_O_2_ has not been fully cognised. It has been demonstrated that H_2_O_2_ application leads to increased sensitivity to strong light in a cyanobacterial mutant that is defective in MC production [1], which suggests a role of MC in the protection against oxidative stress. Rapid initiation of the antioxidant defence followed by a change in the MC content was also observed in *Microcystis aeruginosa* in its response to oxidative stress [2]. Thus, fast reduction of MC concentration by H_2_O_2_ in blooming water bodies has been questioned because the physiological response may include enhanced MC production. On the other hand, recent research on MC involvement in defence against oxidative stress [3] documented that MC-producing strains are less resistant to H_2_O_2_ than non-toxic strains. Further, field experiments have provided other arguments that H_2_O_2_ used alone is not effective in fast MC elimination [4,5]. Moreover, different responses have been observed among cyanobacteria taxa, indicating a possible species–specific adaptation to H_2_O_2_. These examples indicate the need to combine H_2_O_2_ treatment with other techniques which enable the reduction of MC concentration efficiently. Since the use of different chemicals that are capable of MC degradation may be problematic due to their impact on living organisms, one promising alternative could be enzymatic MC degradation. The metalloprotease MlrA, called microcystinase, is a product of the *mlrA* gene that occurs naturally in several bacterial species [6]. It is the only known enzyme that is capable of hydrolysing the stable MC cyclic structure. Heterologous expression and production systems for this enzyme have already been developed [7], including the recently proposed alternative production of this enzyme in autotrophic hosts [8]. Furthermore, recent research [9] provides an indication that the recombinantly produced MlrA should be considered a very effective agent which hydrolyses and detoxifies different MC variants.

The aim of the present study is: (i) to provide more information on how lower H_2_O_2_ doses influence MC production in laboratory conditions; (ii) to test the efficiency of MC linearization (as catalysed by MlrA) in environmentally relevant conditions; (iii) to perform preliminary mesocosm experiments of the combined H_2_O_2_ + MlrA treatment in environmental water (Lake Ludoš, Serbia).

## 2. Results

### 2.1. Laboratory Experiments

#### 2.1.1. MlrA Activity

**Figure 1:**
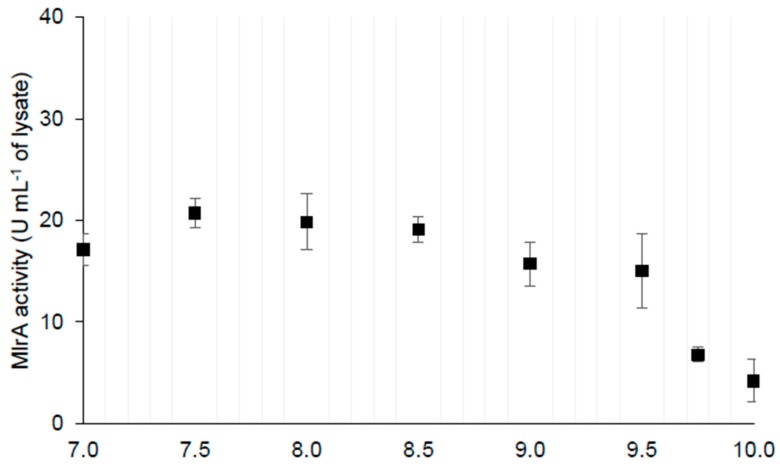
MlrA activity profile in the pH range of 7.0 to 10.0.

To determine the amount of MlrA required to degrade the MC during the mesocosm study, some preliminary analyses were carried out. Table 1 (parts A–D) shows the data on the MlrA activity in lysates and its dependence on crucial parameters. Furthermore, the MlrA activity in different pH levels is shown in Figure 1. In the pH range of 7.00–9.50, the MlrA activity is stable. The activity decreases by about 50% at pH 9.75 and 10.00 (Table 1 and Figure 1). The temperature of the treated lake was another important factor influencing the MlrA activity (i.e., MC degradation efficiency). In comparison with the optimal temperature (about 20 °C), the MlrA activity decreased by 2.4-fold and 4.6-fold at 10 °C and 5 °C, respectively (Table 1C). The MlrA activity in the *M. aeruginosa* culture and in the sample from Lake Ludoš was lower in comparison with the control (Table 1D). On the other hand, the activity was relatively stable for 24 h of incubation.

#### 2.1.2. The Response of *M. aeruginosa* to Lower Doses of H_2_O_2_

Laboratory analyses were performed to determine both the physiological (MC synthesis and its intracellular accumulation) and the transcriptomic response (*mcy* expression) of a MC-producing strain to lower doses of H_2_O_2_ (2 and 3 mg L^−1^, treatment three times per week).

**Figure 2:**
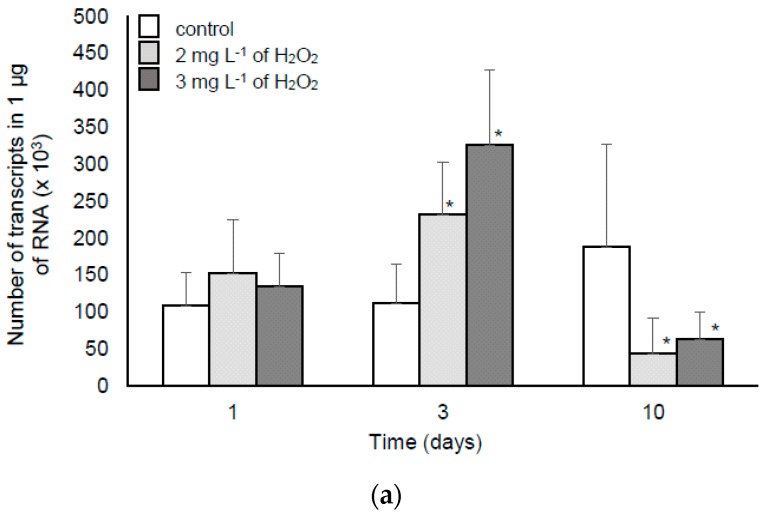

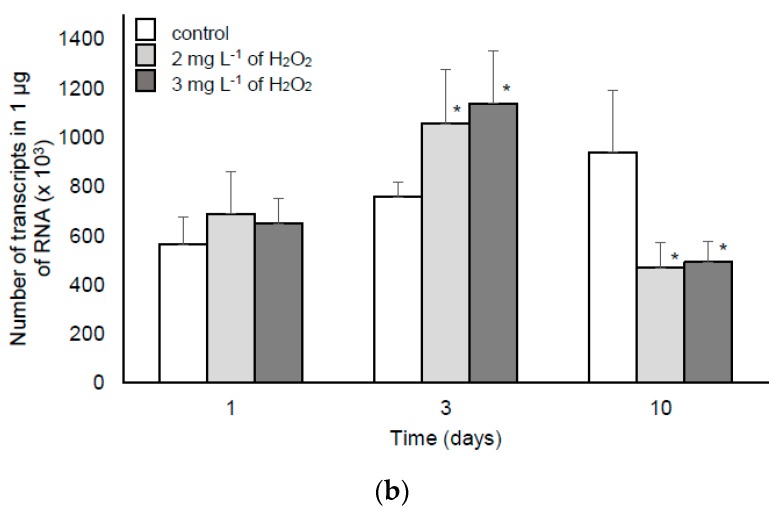
Level of *mcyB* (**a**) and *mcyE* (**b**) transcripts of *M. aeruginosa* PCC 7813 after incubation with H_2_O_2_. The white blocks represent the control culture, whereas the light-grey and dark-grey blocks represent *M. aeruginosa* treated with 2 and 3 mg L^−1^ of H_2_O_2_, respectively. Error bars indicate the standard deviation. The asterisk indicates statistically significant differences in comparison with the control (n = 3, *p* < 0.05).

**Figure 3:**
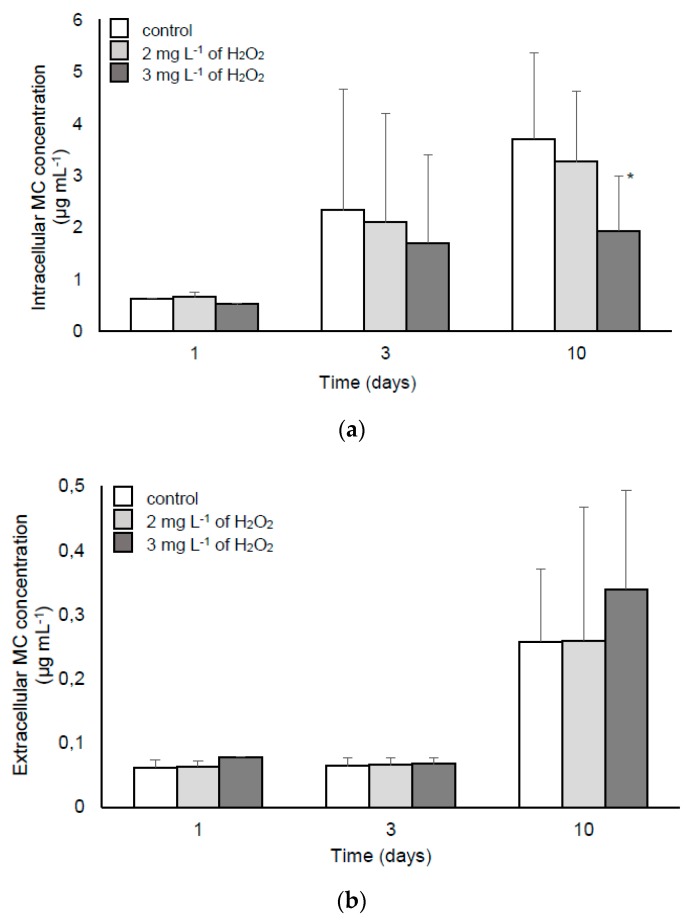
Intracellular (**a**) and extracellular (**b**) MC content in the *M. aeruginosa* PCC 7813 culture after incubation with H_2_O_2_. The white blocks represent the control culture, whereas the light-grey and dark-grey blocks represent cultures treated with 2 and 3 mg L^−1^ of H_2_O_2_, respectively. The error bars indicate the standard deviation. The asterisk indicates statistically significant differences in comparison with the control ((n = 3, *p* < 0.05).

The expression of *mcyB* and *mcyE* genes as well as MC content are shown in Figure 2 and Figure 3, respectively. The growth of H_2_O_2_-treated cyanobacteria was slightly reduced only in the culture treated with 3 mg L^−1^ of H_2_O_2_ (Figure S1). Both applied doses of H_2_O_2_ (2 and 3 mg L^−1^) induced a higher expression of *mcyB* and *mcyE* genes on the third day of the experiment. On the 10th day, a decrease of transcripts was noted in comparison with the control (Figure 2). However, the extracellular MC content (Figure 3b) was not affected by H_2_O_2_, whereas in the presence of 2 and 3 mg L^−1^ of H_2_O_2_, the intracellular MC content was lowered (Figure 3a and Figure S1).

### 2.2. Field Experiments

The preliminary measurements were conducted to determine temperature, pH, and MC concentration in the lake, which was necessary to verify if the conditions were appropriate for the commencement of the mesocosm experiment (Table 2). The overall condition of the Lake indicated a poor chemical and ecological status, which was a reason why it was selected for the mesocosm experiment. Some chemo-physical parameters (pH >8 and high O_2_ saturation) suggested high CO_2_ absorption and O_2_ evolution, i.e., a high photosynthesis activity. Furthermore, the electrical conductivity was relatively high. Studies described above (Section 2.1) allowed for planning the main field experiment, which aimed to analyse the impact of different treatments on the investigated reservoir. The short-term effect of the three different treatments (group A—control; B—treatment with H_2_O_2_; C-H_2_O_2_ and MlrA; D-MlrA, see the details in Section 2.2) on the phytoplankton community structure, MC concentration, and the expression of genes involved in MC synthesis was analysed. Two independent stages allowed for determining the treatment efficiency during a natural development of the phytoplankton community.

#### 2.2.1. Phytoplankton Content in Containers

Table 3 and Table 4 show the influence of treatments on phytoplankton content in containers. In the first stage of the experiment (Table 3), the total cyanobacteria abundance decreased in two experimental groups (B, C) in comparison to group A (without any treatments) and the initial abundance that was measured a few days before the experiment (time zero). The most dramatic decline in abundance was observed in group B (treated with H_2_O_2_ only)_._ Among cyanobacteria, the most abundant were *Limnothrix* sp., *A. flos-aquae*, and *Planktothrix agardhii* (Table S2). Decreased abundance of all the three taxa was observed in all three treatments (except *Limnothrix* sp. in group D), with the highest decline in group B. At the same time, the abundance of eukaryotic phytoplankton was relatively stable and decreased slightly only in group B. The most numerous taxa within the eukaryotic algae were *Cyclotella* sp., *Monoraphidium contortum*, and *Tetradesmus lagerheimii* (Table S2).

In the second stage (Table 4), the total cyanobacteria abundance decreased in group C (treated with H_2_O_2_ and MlrA), while in group D, the abundance increased in comparison to group A (without any treatments) and the initial abundance that was measured a few days before the experiment (time zero). In this group, the most abundant were *P. agardhii*, *Jaaginema sp*., and *A. flos-aque* (Table S3). A decline in the abundance of the latter group was noted in all three treatments, whereas the content of *P. agardhii* was lower only in groups B and C and was higher in group D.

On the other hand, an increased abundance of *Jaaginema* sp. in group B was observed. The number of MC-producing cyanobacteria, primarily *P. agardhii,* was higher than in the first stage of the study. The abundance of eukaryotic phytoplankton decreased slightly in group C. *Monoraphidium contortun*, *Cyclotella* sp., and *Desmodesmus* spp. were the most abundant taxa within this group (S4).

#### 2.2.2. MC Synthesis, Extra- and Intra-cellular MC Concentration in the Samples

The RNA isolation from the field samples was performed according to standard protocols. However, the quantitative analysis of *mcy* gene expression in the containers was not possible due to the lack of fluorescence signal, which was probably the outcome of some unidentified quenching originating from the environment. A semi-quantitative comparison of the transcripts level did not allow for observing any dramatic differences between the four experimental groups (data not presented).

**Figure 4:**
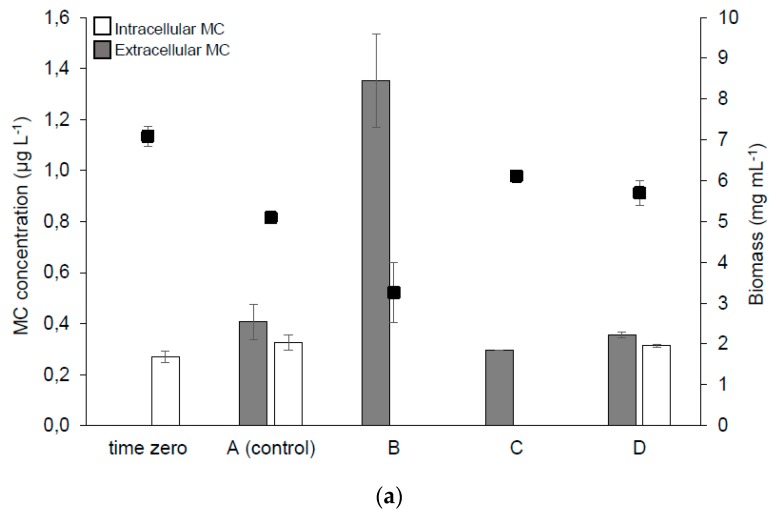

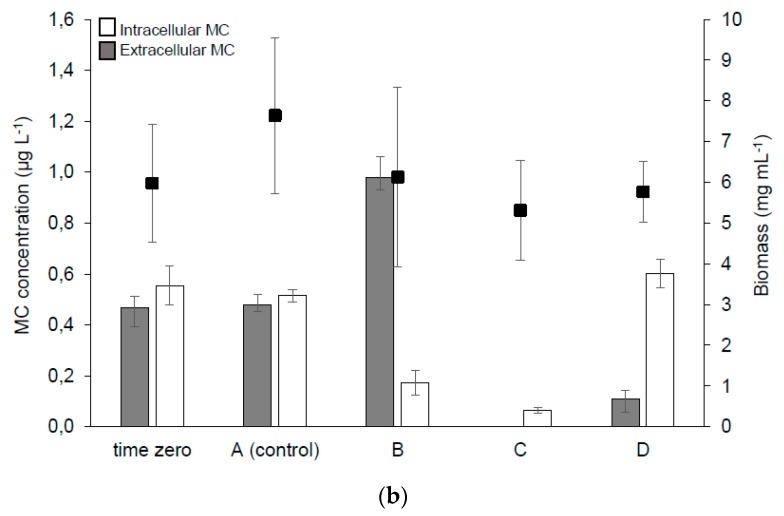
MC concentration in containers during stage I (**a**) and stage II (**b**) of the mesocosm experiment. The white and grey blocks represent intra- and extra-cellular MC, respectively, whereas the black squares stand for the biomass production. A stands for control, B—H_2_O_2_, C—H_2_O_2_ + MlrA, D—MlrA. The parameters were determined before the treatment (time zero), on the sixth day (stage I), and the third day (stage II) of the treatment (groups A–D). The error bars indicate standard deviation (n = 4). Statistically significant differences (*p* < 0.05) in total MC (extra- and intra-cellular) in both stages were found between: A versus C; B versus C and D; C versus D; C versus D.

Preliminary measurements (of 16 March 2018) using the ELISA assay showed the presence of both intra- and extra-cellular MC fractions (Table 2). Thus, the first stage of the experiment began in early April. Intra- and extra-cellular MC concentrations in the samples from containers are presented in Figure 4. HPLC analyses documented the presence of a dmMC-RR variant in the samples collected before and after treatment (Figure 4a), which was in line with previous findings that this MC variant was present in the investigated lake [10]. Total MC was below the provisional guideline value for drinking water (1 µg L^−1^) at the beginning of the experiment (time zero) and in control group A after 5 days. The addition of H_2_O_2_ only (group B) resulted in a significant increase in the dmMC-RR concentration in the extracellular fraction in comparison with group A, whereas its intracellular fraction was almost eliminated due to cell lysis. MlrA supplementation (groups C and D) allowed for bringing down the dmMC-RR level to below 0.4 and 0.7 µg l^−1^, respectively. In the second stage of the experiment (Figure 4b), treatment with H_2_O_2_ (group B) caused an effect similar to the one observed in April, however the efficiency of MC elimination after combined treatment (H_2_O_2_ + MlrA, group C) was even better and the extracellular fraction of dmMC-RR was degraded almost completely.

## 3. Discussion

Previous preliminary in vitro experiments using samples from Lake Ludoš put into question the efficiency of hydrogen peroxide in treating cyanobacteria in a dense bloom [10]. The recommended doses of H_2_O_2_ (up to 5 mg L^−1^) caused only a slight viability inhibition of the cyanobacteria. Additionally, the intracellular MC concentration was reduced only after the application of a dose of 20 mg L^−1^ of hydrogen peroxide. It was suggested that doses of 2 and 5 mg L^−1^ may even stimulate MC production. However, the authors suggested that the H_2_O_2_ treatment could be more effective in spring when the MC-producing species initialize their growth and thus, stop the domination of these cyanobacterial species in warmer periods. Furthermore, as reported by Schöne et al. [11], toxic *Microcystis* cells which migrate in the spring from the sediments into the surface water layers have an advantage because of radical scavenging by MC. This phenomenon may promote the selection of a subpopulation with a higher MC production. These arguments were considered strong reasons to start the mesocosm experiments in April.

### 3.1. The Impact of H_2_O_2_ on the MC Production by M. aeruginosa

Several recent reports have provided information on the protective role of MC against oxidative stress (including that caused by H_2_O_2_) by binding covalently with redox-sensitive proteins and thus, preventing their proteolytic degradation [1,12]. Some external or internal stimulation may cause both an increased content of intracellular MC per cell and up-regulation of *mcyE* gene expression [13]. On the other hand, Schuurmans et al. [3] hypothesised that MC may suppress the activity of proteins involved in H_2_O_2_ degradation. Furthermore, some opposite conclusions have been presented regarding the impact of H_2_O_2_ on MC production by toxic strains. Low, environmentally relevant concentrations of H_2_O_2_ may induce MC production that is necessary for the protection against such mild oxidative stress [1], however higher H_2_O_2_ doses result in a decrease in the total (extra- and intra-cellular) MC content [3]. As suggested previously [10], 2–5 mg L^−1^ of H_2_O_2_ is probably too little to induce cyanobacterial lysis in dense blooms and can stimulate the accumulation of cell-bound (intracellular) MC as a reaction to oxidative stress. According to these data, it is still an open question what H_2_O_2_ doses (typically between 2 and 10 mg L^−1^) should be recommended to eliminate toxic cyanobacteria (and ideally cyanotoxins) but at the same time to cause a minimal impact on other biota. Schuurmans et al. [3] suggested that the dose should depend on the initial cell density in order to achieve a necessary level per cell.

Previous research indicated that the dose 5 mg L^−1^ of H_2_O_2_ caused extensive cell lysis of the *M. aeruginosa* culture within a few days [9] and thereby, such a treatment cannot stimulate MC production. To verify the impact of somewhat lower doses on MC synthesis and its intracellular accumulation, we analysed *mcy* expression as well as MC content in the *M. aeruginosa* laboratory culture treated repeatedly with 2 and 3 mg L^−1^ of H_2_O_2_. The changed level of *mcy* transcripts provided another confirmation that the MC synthesis pathway could be influenced in given conditions (Figure 2). Schuurmans et al. [3] documented the downregulation of MC genes after 24 h H_2_O_2_ stress. Our results were partially contrary to this finding. An upregulation was observed on the third day of incubation with H_2_O_2_, which suggests that after short exposure to oxidative stress, the cells tried to respond by a higher MC production. On the other hand, repeated supplementation of the culture with H_2_O_2_ (10 days of exposure) could suppress the metabolic activity, which resulted in the downregulation of *mcyB* and *mcyE* expression (Figure 2). However, the documented MC concentration did not simply reflect the changed *mcyB* and *mcyE* levels and there was no significantly higher MC production (Figure 3). This shows that despite some increase in the transcript level, the concentration of H_2_O_2_ in the range of 2–5 mg L^−1^ should be safe in the context of MC production. However, the differences observed in some studies [1,3] indicate that the used H_2_O_2_ concentration and the length of exposure are of crucial importance for cell lysis and other observed effects. 

### 3.2. The Efficiency of MlrA in Environmentally Relevant Conditions

As was mentioned above, the treatment of cyanobacteria with hydrogen peroxide should be supplemented with other agents (e.g., MlrA). Such an arrangement not only suppresses the growth of cyanobacteria or induces their death, but also enables a simultaneous, fast degradation of the MC released from the cells disrupted by the algicide. Recently, some new methods of biological MC degradation have been presented [14,15,16], however these proposals only offer ex situ treatment of contaminated water in bioreactors designed for MC removal from filtered water. Another approach is in situ bioremediation, which requires the production of an enzyme that is capable of MC degradation by well-established heterologous systems and the release of the enzyme into the environment. Such an enzyme-based bioremediation represents a simple, fast, and ecologically acceptable approach [17]. Microbial enzymes have been used instead of whole cells to overcome the main limitations of bioremediation based on microorganisms, i.e., the degradation rate [18].

Some of the crucial parameters influencing MlrA activity and stability have been studied previously [7]. In the current paper, some additional analyses were carried out to determine the amount of MlrA required to degrade MC in the containers during the mesocosm study. A novel expression system (using *E. coli* C41(DE3) as a host) allowed for more efficient MlrA production in comparison with the previously established methods. SDS/PAGE of the total cell lysates of *E. coli* C41 before and after induction with IPTG demonstrated the overexpression of the recombinant MlrA. The average activity of recombinant MlrA against MC-LR in lysate (Table 1) was significantly higher in comparison with the lysates from the previous expression systems, compared in a paper by Dexter et al. [8]. Thus, the use of this system of MlrA production for field study and further applications is more justified and efficient. Furthermore, it has been documented that the specificity of MlrA is very high and the only known molecules hydrolysed by this enzyme are MC and NOD [7]. Thus, MlrA seems to be a very neutral agent that should not impact other aquatic organisms.

Previously [9], MlrA activity was documented as stable in the presence of H_2_O_2_. Other crucial data were also described in Section 3.1: the MlrA activity at different pH, temperature, and in lake water samples. Additionally, some of the physico-chemical parameters of Lake Ludoš measured at the beginning of the subsequent stages (pH, temperature, and the total MC content extra- and intra-cellular, Table 2) were taken into account in designing the experimental setting. In particular, the reduced MlrA activity in lake water versus the activity in buffer and lower MlrA activity at 10 °C (important during the first step of the experiment) necessitated the use of a higher volume of the enzyme extract. The stability of MlrA activity within 24 h of incubation was one of the important findings that enabled the application of the combined treatment. Based on these data, the MlrA amount required for complete MC degradation was calculated (Table 1). 

### 3.3. Treatment in Lake Ludoš

A successful application of H_2_O_2_ against cyanobacteria has been shown in many studies [5,19,20] since H_2_O_2_ was first proposed as an effective algicide by Kay et al. [21]. However, many studies only examined H_2_O_2_ toxicity in the MC-producing species from *Planktothrix* and *Microcystis* genera. Much less is known on the interspecific variation across other common bloom-forming and potentially toxic cyanobacteria from the genera *Dolichospermum, Aphanizomenon*, or *Raphidiopsis*, however a wide range of H_2_O_2_ toxicity thresholds against these cyanobacteria have been documented [22,23,24]. The current study has shown different responses of cyanobacteria to the treatment, depending on the stage of the experiment. Moreover, the impact of the treatments varied among dominant cyanobacteria. A decreased abundance of cyanobacteria in groups with H_2_O_2_ (B and C) in stage I was expected as a typical effect resulting from weak resistance of cyanobacteria to H_2_O_2_. What is particularly important is that MC-producers reacted in the same way (Table 3 and Table 4). An unusual effect was observed in the second stage; H_2_O_2_ alone did not cause a reduction of total cyanobacterial abundance, but H_2_O_2_ + MlrA treatment significantly decreased the level of cyanobacteria (Table 3 and Table 4). However, excluding non-toxic *Jaaginema* sp. (which grew well in the presence of H_2_O_2_), a significant reduction of other cyanobacteria, including the potential MC-producers (*P. agardhii* and *A. flos-aquae*), was observed in groups B and C alike. This is partly in line with studies of Schuurmans et al. [3], documenting that non-toxic strains rapidly degraded H_2_O_2_ and subsequently recovered in contrast to toxic strains. Higher resistance of *Jaaginema* sp. to H_2_O_2_ is another example that the response of different cyanobacteria to H_2_O_2_ may vary. In line with earlier studies [20,25], we observed a relatively stable level of eukaryotic algae in both stages of the experiment. An increasing abundance of green algae and a decreasing abundance of diatoms could be related to the spring season and increasing water temperature favoring the development of green algae. These ambiguous results indicate how unpredictable and diverse effects may be observed when applying the H_2_O_2_ treatment and that several factors (e.g., the phytoplankton composition or season) may influence the final effect. 

Recent research has clearly demonstrated that the treatment of *M. aeruginosa* culture with H_2_O_2_ only (dose 5 mg L^−1^) caused (by cell lysis) a fast increase of the extracellular MC content and that such a treatment is not satisfactory in the short-term perspective because of the MC release but no subsequent degradation of these compounds. The intracellular MC content decreased due to the cell lysis, whereas the extracellular MC concentration increased after several days of treatment with H_2_O_2_ in both studies. The current mesocosm study in Lake Ludoš (inhabited by MC-producers *Planktothrix* sp. and *Microcystis* sp.) has provided very similar conclusions (Figure 4). In contrast, a field experiment by Yang et al. [5] and the laboratory study of Fan et al. [26] indicated a significant decrease of both extra- and intra-cellular MC fractions (7 and 3 days after treatment with 6.7 and 10.2 mg L^−1^ of H_2_O_2_, respectively). Thus, depending on the investigated cyanobacterial strains (or natural populations), the results may differ significantly and the final effect of treatment may vary. The use of higher H_2_O_2_ doses (10–20 mg L^−1^), even in the case of dense cyanobacterial populations, is questionable because of its negative impact on zooplankton viability [25]. This clearly points to the application of combined treatment. In the previous paper [9], the calculation of the required amount of enzymes necessary to purify a given volume of water was presented. Based on the newest data (Table 1), some corrections are necessary, taking into account the new expression system (which allow for producing more MlrA), however also the reduced MlrA activity in the lake water. Efficient purification of a 5000 m^3^ fish pond contaminated with 5 μg L^–1^ of MC would require approximately 270 L of *E. coli* culture (in other words, 137,000 Units).

We are postulating that only the H_2_O_2_ + MlrA treatment proves to be effective in the reduction of both toxic cyanobacteria (Table 3, Table 4, Tables S2 and S3) and the MC concentration (Figure 4). Such a combined treatment offers expected improvement of freshwater composition, which could be particularly important when the MC concentration must be reduced very quickly. Another interesting observation of a possible MlrA impact was documented in the second stage of the experiment—the treatment with MlrA (group D) increased the content of all the main cyanobacteria, including the MC-producers (Table 3 and Table 4). This observation suggests that the MC degradation (by MlrA) may favor the growth of cyanobacteria. However, to confirm such a hypothesis, further studies are required.

## 4. Conclusions

The aim of this paper was to establish a new “in situ” method for the elimination of cyanobacteria and MC. It was assumed that the potential of H_2_O_2_ in suppressing a cyanobacterial bloom could be used in combination with an enzyme (MlrA) which degrades MC. Such an arrangement would not only result in growth suppression or death of cyanobacteria, but also in enhanced and fast enzymatic degradation of MC released from the cells lysed by H_2_O_2_. After laboratory experiments and field work, the following conclusion may be formulated: a) the enzyme activity depends on environmental conditions, especially on pH and temperature; b) experimental conditions of 20 °C, pH 7.0–9.5, 5 mg L^−1^ of H_2_O_2_, 14 Units of MlrA (per 100 L contaminated with 10 µg MC L^−1^) were found to provide efficient elimination of both cyanobacteria and MC in lake water. Cyanobacteria were significantly more sensitive to H_2_O_2_ than other phytoplankton. This new concept, of the combined treatment with both H_2_O_2_ and MlrA enzyme, is proposed to be applied in conditions where cyanobacterial biomass with a high MC concentration needs to be rapidly and efficiently reduced.

## 5. Materials and Methods 

### 5.1. Chemicals and Strains

Trifluoroacetic acid (TFA) was purchased from Sigma (St Louis, MO, USA), hydrogen peroxide was obtained from Krakchemia S.A. (Kraków, Poland), and the Pursuit 3 C18 column and acetonitrile (ACN) were obtained from Agilent (Santa Clara, CA, USA). Standards of MC were extracted and purified from a culture of the *Microcystis aeruginosa* PCC 7813 strain (the Pasteur Institute, Paris, France) and from the *Microcystis* NIES 107 strain. 

*Escherichia coli* C41(DE3) (Novagen Madison, WI, USA), which is effective in producing membrane and toxic proteins [27], was used to express C-terminal his-tagged MlrA from pET21a-mlrA plasmid. For the expression of recombinant protein, the strain was grown at 37 °C in LB broth supplemented with ampicillin (100 µg mL^−1^). The *M. aeruginosa* PCC 7813 strain was cultivated in a Z-medium [28] at 20 °C in 40 µmol m^−2^ s^−1^ of photosynthetically active radiation (provided in a light/dark cycle; 12/12 h).

### 5.2. MlrA Assays

The lysate of a heterologous *E. coli* C41(DE3) /pET21a-mlrA was obtained by sonication with an ultrasonic processor UP100H (Hielscher Ultrasonics GmbH, Teltow, Germany). The centrifuged lysates were used as a source of the MlrA enzyme. MlrA activity assays were performed in the following manner: 5 µL of the enzyme in different dilutions was added to 45 µL of the MC solution. The enzyme and MC were suspended in a PBS buffer, pH 7.0. The final MC concentration was 1 µg ml^−1^. The incubation temperature was 20 °C and the reaction was stopped after 1 h by the addition of 5 µl of 1% TFA. Samples were cooled to 5 °C and analysed by HPLC. In longer experiments, the volumes of both enzyme and reaction mixtures were bigger and the samples for HPLC were collected after subsequent times of incubation. As the lake water that was used in the mesocosm experiment was alkaline (Table 1), the MC-LR hydrolysis was assessed in the range 7.00–10.00, with 0.25 steps to check the impact of higher pH on the MlrA activity. Recombinant MlrA was diluted in 0.12 M Britton Robinson buffer prepared with equimolar amounts of acetic, phosphate, and boric acids co-dissolved and adjusted to a given pH. The possible impact of MC producers as well as biotic and abiotic factors on the MC degradation efficiency was verified by an activity assay with *M. aeruginosa* cultures and samples from Lake Ludoš.

### 5.3. M. aeruginosa Cultivation and Treatment with H_2_O_2_

Three independent *Microcystis aeruginosa* PCC 7813 cultures (volume 50 mL, initial density OD_730_ = 0.2, each in triplicate) were grown at 20 °C with BG11 medium, under 30 µmol photons m^−2^ s^−1^. H_2_O_2_ was added at the beginning of the experiment and then every 3 days in the final concentration of 0, 2, and 3 mg L^−1^ for groups A (control), B, and C, respectively. On days 1, 3, and 10, the samples were collected for: OD_730_ measurement, RNA isolation, and the determination of extra- and intra-cellular MC concentration. Samples for RNA isolation were prepared as follows: 4.5 mL of ethanol was added immediately to 9 mL of the culture and centrifuged for 7 min at 14,000 rpm. The pellet was frozen in liquid nitrogen and stored at −20 °C. MC extraction from 1 mL of culture was performed using the procedure described by Meriluoto and Spoof [29]. Centrifuged cells were frozen and thawed twice, the cells were then ultrasonicated in 0.3 mL of 75% methanol and after centrifugation, the supernatant was evaporated under argon. The evaporated samples were diluted in 200 µL of 75% methanol. Extracellular MC was determined directly in the medium. 

### 5.4. Mesocosm Experiment in Lake Ludoš

Lake Ludoš is a shallow lake in northern Serbia. It was chosen as the experimental site because of its permanent eutrophic state and almost constant cyanobacterial blooming, resulting in the presence of toxic cyanobacteria, MC release, and MC accumulation in freshwater organisms [10]. A detailed description of the main lake parameters may be found in Tokodi et al. [10]. Sixteen plastic containers stabilized by a wooden frame were installed at the center of the lake (46.102159 N, 19.821149 E) on April 4, 2018. The containers were 0.3 m × 0.3 m × 0.48 m (height) with a volume of about 43 L. The top of the containers were adjusted to about 10 cm above the water surface. Additionally, the containers were shielded by a 20 cm high plastic wall to protect them from the waves and were covered with a plastic net to protect them from birds and other animals. Before the treatment (time zero), the samples were collected from the containers and initial phytoplankton composition and MC content were analysed. Then, four experimental groups were established in four replicates: A—control; B—water treated with H_2_O_2_ (5 mg L^−1^ final concentration); C—water treated with H_2_O_2_ (5 mg L^−1^) and MlrA; D—water treated with MlrA. 3.3 mL of 50% H_2_O_2_ were diluted in 5 L of water from the lake and 620 mL of the diluted H_2_O_2_ were added to eight containers (groups B and C). MlrA was diluted 1000 times in lake water, then 130 mL of the diluted enzyme (which corresponds to 5.4 Units) was added to containers C and D. Water in the containers was mixed manually. During the first stage of the experiment, the samples (1 L) were collected on the first day and after 6 days. A second independent experiment began on 23^rd^ April and the samples were collected on the first day and after 3 days. Due to the higher temperature during the second stage, only 1.2 Units of MlrA was added to containers C and D.

### 5.5. Chemo-physical and Biological Parameters of Lake Ludoš

Chemo-physical parameters of the water were measured on 16th March 2018, before the mesocosm experiment. Measurements in the field (temperature, pH, conductivity, O_2_ concentration, and O2 saturation) were performed using multi-parameter probes (Xylem Analytics, Weilheim, Germany). TSS (total suspended solids), TOC (total organic carbon), NO_3_, detergents, COD (chemical oxygen demand), and BOD (biological oxygen demand) were measured in the laboratory with a Pastel UV Secomam.

In order to determine the production of biomass in containers, 100 mL of water was filtered through Whatman GF/C filters and chlorophyll *a* was determined according to Mackinney [30]. The measurements were done in duplicate and the results were expressed as means. The biomass production was calculated using an indirect method [31].

Water samples for a phytoplankton analysis to assess the abundance of cyanobacteria and other groups of phytoplankton were collected directly before and after treatments from the surface layer of all four types of containers and were preserved with Lugol solution. The samples were sedimented in a 1 L glass cylinder for 48 h. The overlying water was decanted off and a volume of about 30–50 mL was used for the phytoplankton analysis. Species identification and counts were conducted using an Olympus light microscope under × 400 magnification. The phytoplankton enumeration was carried out in 100–150 fields of a Fuchs-Rosenthal chamber, which ensured that at least 400 specimens were counted to reduce the error to less than 10%. A single cell, a coenobium, or a filament represented one specimen in the analysis. 

The ELISA assay (according to the provided protocol-Microcystin/Nodularin ADDA ELISA, Beacon, NY, USA) was used for a preliminary determination of MC concentration (16 March 2018) in order to prepare adequate volumes of MlrA for the following mesocosm experiment.

Sample preparation and HPLC determination of MC concentration were done using the procedure described by Meriluoto and Spoof [29]. Briefly, biomass collected on Whatman GF/C filters after filtering 250 mL of lake water was frozen and thawed twice, the cells were ultrasonicated in 3 mL of 75% methanol, and after centrifugation, the supernatant was evaporated under argon. To determine the extracellular MC in samples from containers, 350 mL of filtered water was passed through the solid phase extraction cartridge and the MC were eluted with 3 mL of acetonitrile containing 0.05% TFA. Solvent was evaporated under argon. The evaporated samples were diluted in 200 µL of 75% methanol and MC concentration was determined using an Agilent 1220 Infinity Gradient DAD LC System. The HPLC runs were performed as previously described [32]. The presence of MC-LR, dmMC-LR, MC-RR, dmMC-RR, MC-YR, MC-LW, and MC-LF variants was monitored.

### 5.6. Real Time PCR

Cyanobacterial RNA isolation was carried out from 90 mL of sample centrifuged in 50 mL falcon tubes (5000 rpm, 5 °C, 12 min) using TRI Reagent (Molecular Research Center, Cincinnati, OH, USA) according to the manufacturer’s protocol. 

In order to quantify mRNA with qPCR, *mcyB*_M2q and *mcyE*_M2q primers (Table S1) were designed from the *mcyB* and *mcyE* genes sequences of *M. aeruginosa* deposited in the Nucleotide NCBI database using the RealTime PCR Tool software (https://eu.idtdna.com/scitools/Applications/RealTimePCR). The sequences were verified using the OligoAnalyzer software (https://eu.idtdna.com/calc/analyzer) to avoid the formation of secondary structures. 1000 ng of RNA, quantified with a spectrophotometer (NanoDrop Technologies, Wilmington, DE, USA), were used for cDNA synthesis using the High-Capacity cDNA Reverse Transcription Kit (Applied Biosystems, Foster City, CA, USA) as per the manufacturer’s instructions. Real-time PCR was performed in duplicate (three biological replicates) using the CFX96 Touch Real-Time PCR Detection System (Bio-Rad, Hercules, CA, USA) and the PowerUp SYBR Green Master Mix (Thermo Scientific, Waltham, MA, USA).

The real-time PCR programme was as follows: an UDG activation step of 2 min at 50 °C, a Dual-Lock DNA polymerase activation step of 2 min at 95 °C, and 40 cycles of 15 s at 95 °C and 60 s at 60 °C. An end-point melt-curve analysis was generated after each run and was analysed to assure the absence of nonspecific PCR products. 

A 10-fold dilution series of linearized pJET1.2 plasmids containing *mcyB* and *mcyE* from *M. aeruginosa* were used for standard curves [33]. The plasmids were prepared by cloning the amplicons of the *mcyB* and *mcyE* genes fragments using the CloneJET PCR Cloning Kit (Thermo Scientific) and linearized by FastDigest NotI restriction enzyme (Thermo Scientific). Amplicons were prepared with *mcyB*_M2q and *mcyE*_M2q primers. The cDNA quantities in the samples (hence mRNA) were calculated using the linear regression equations of the standard curves for each assay.

Semi-quantitative PCR was performed using the DreamTaq DNA Polymerase (Thermo Scientific). The real-time PCR programme was as follows: an initial denaturation step of 3 min at 95 °C, and 25–34 cycles of 30 s at 95 °C, 30 s at 62 °C, and 60 s at 72 °C. The *rp°C1* sequence was used as a standard.

### 5.7. Statistical Analysis

The repeated measures ANOVA (Statistica 10) and Tukey test were used to analyse the statistically significant differences between the experimental groups in phytoplankton abundance, the level of *mcy* transcripts, and the MC content in *M. aeruginosa* cultures.

## Figures and Tables

**Table 1:** MlrA production efficiency, its activity in different conditions, and the calculated amount of enzyme necessary to degrade the Microcystins (MC) in the mesocosm experiment. The asterisk indicates a statistically significant difference in comparison with the value from the range of 7.0–9.5 pH (*p* < 0.01).

**A. Production efficiency**	average MlrA activity after isolation from *E. coli* C41	mU µL^−1^ of lysate	U mL^−1^ of culture		
		41.6 ± 24.0	1.2 ± 0.7		
**B. Dependence on pH**	MlrA activity in different pH (U mL^−1^ of sample)	activity range in pH 7.0–9.5	average activity in pH 7.0–9.5 17.9 ± 2.3	average activity in pH 9.75–10.00 6.8 ± 4.2 *	
		15.07–20.76			
**C. Dependence on temperature**	MlrA activity in different temperatures (U mL^−1^ of sample) percentage in comparison with 20 °C	20 °C	10 °C	5 °C 2.9 21.9	
		13.4	5.5 40.9		
	fold the reduction in comparison with 20 °C		3.9	5.0	
**D. Activity in the culture and sample from the lake**		control	1 h	3 h	24 h
	MlrA activity (U mL^−1^) in *M. aeruginosa* culture OD_730_ = 0.2	49.2	9.6 ± 1.2	20.8 ± 10.2	5.4 ± 2.6
	MlrA activity (U mL^−1^) in *M. aeruginosa* culture OD_730_ = 0.5	49.2	8.5 ± 2.2	15.3 ± 5.4	7.4 ± 4.4
	MlrA activity (U mL^−1^) in the sample from Lake Ludoš (collected in 16.03.2018)	49.2	3.0 ± 0.8	2.5 ± 0.2	2.8 ± 0.7
**E. Amount of MlrA (Units) required for complete MC degradation in 100 l (initial MC concentration 2 µg L^−1^)**		Optimal conditions 0.16	L. Ludoš, stage I 12.6	L. Ludoš, stage II 2.8	

**Table 2:** Chemo-physical parameters and MC content of Lake Ludoš before the mesocosm experiment.

Parameters	16 March 2018	
	Pier	Center of the Lake
	(46.103207 N, 19.821360 E)	(46.102159 N, 19.821149 E)
temperature (°C), in situ	10	10.1
pH, in situ	8.3	8.3
concentration O_2_, in situ (μg mL^−1^)	13.78	18.56
saturation O_2_, in situ (%)	129.8	166.6
conductivity, in situ (µS cm^−1^)	875	872
total suspended solids (TSS) (mg dm^−3^)	47.0	39.0
total organic carbon (TOC) (mg dm^−3^)	8.5	8.4
NO_3_ (mg dm^−3^)	≤0.5	≤0.5
detergents (mg dm^−3^)	2.1	2.0
chemical oxygen demand (COD) (mgO_2_ dm^−3^)	24.6	23.1
biological oxygen demand (BOD) (mgO_2_ dm^−3^)	12.1	11.9
**MC content** (μg L^−1^)		
intracellular MC content	1.67	1.84
extracellular content	1.31	1.22
total	1.55	1.57
unprepared	1.49	1.53

**Table 3:** The response of cyanobacteria and other phytoplankton to the treatment at stage I of the mesocosm experiment. Potential MC-producers are indicated by italic. * or ** refer to statistically significant differences (*p* < 0.05) in comparison with time zero (before the experiment) or control, respectively. A stands for control, B—H_2_O_2_, C—H_2_O_2_ + MlrA, D—MlrA.

Phytoplankton	Time Zero	A (Control)	B	C	D
Cyanobacteria (cells mL^−1^)	10,620	17013 *	380 **	5400 **	15100
percentage in comparison with group A			2	32	89
percentage of the whole phytoplankton	23	44	2	18	39
*MC-producers (cells mL^−1^)*	*100*	893 *	60 **	120 **	220 **
*percentage in comparison with group A*			*7*	*13*	*25*
*% MC-producers within cyanobacteria*	*1*	*5*	*16*	*2*	*1*
Eukaryotic phytoplankton (cells mL^−1^)	36,180	21,520 *	16,560 **	23,840	23,620
percentage in comparison with group A			77	111	110

**Table 4:** The response of cyanobacteria and other phytoplankton to the treatment at stage II of the mesocosm experiment. Potential MC-producers are indicated by italic. * or ** refer to statistically significant differences (*p* < 0.05) in comparison with time zero (before the experiment) or control, respectively. A stands for control, B—H_2_O_2_, C—H_2_O_2_-MlrA, D—MlrA.

Phytoplankton	Time Zero	A (control)	B	C	D
Cyanobacteria (cells mL^−1^)	2054	2277	2677	569 **	3610 **
percentage in comparison with group A			118	25	159
percentage of the whole phytoplankton	19	14	15	6	22
Cyanobacteria, excluding *Jaaginema sp.*	1985	1554	846 **	354 **	3487 **
percentage in comparison with group A			55	23	224
*MC-producers (cells mL^−1^)*	*1954*	*1338*	723 **	200 **	3364 **
*percentage in comparison with group A*			*54*	*15*	*251*
*% of MC-producers within cyanobacteria*	*95*	*59*	*27*	*35*	*93*
Eukaryotic phytoplankton (cells mL^−1^)	8592	14,092 *	14,985	9262	12,779
percentage in comparison with group A			106	66	91

## Supplementary Materials

The following are available online at https://www.mdpi.com/2072-6651/11/12/725/s1, Table S1: *mcyB*_M2q and *mcyE*_M2q primers, Figure S1: The growth of H_2_O_2_-treated *M. aeruginosa* culture (a) and the intracellular amount of MC calculated per OD_730_ = 1 (b). Table S2: Phytoplankton abundance at stage I of the mesocosm experiment. Potential MC-producers are indicated by the red font, Table S3: Phytoplankton abundance at stage II of the mesocosm experiment. Potential MC-producers are indicated by the red font.
