# Supplementary material for: The Effect of a Combined Hydrogen Peroxide-MlrA Treatment on the Phytoplankton Community and Microcystin Concentrations in a Mesocosm Experiment in Lake Ludoš
Source: Toxins (Basel). 2019 Dec 11;11(12):725. doi: 10.3390/toxins11120725 (PMC6950535; doi:10.3390/toxins11120725)

Figure S1 a. The growth of H_2O_2-treated *M. aeruginosa* culture. The white blocks represent the control culture, whereas the light-grey and dark-grey blocks represent cultures treated with 2 and 3 mg l^{-1} of H_2O_2, respectively. The errors bars indicate standard deviation.

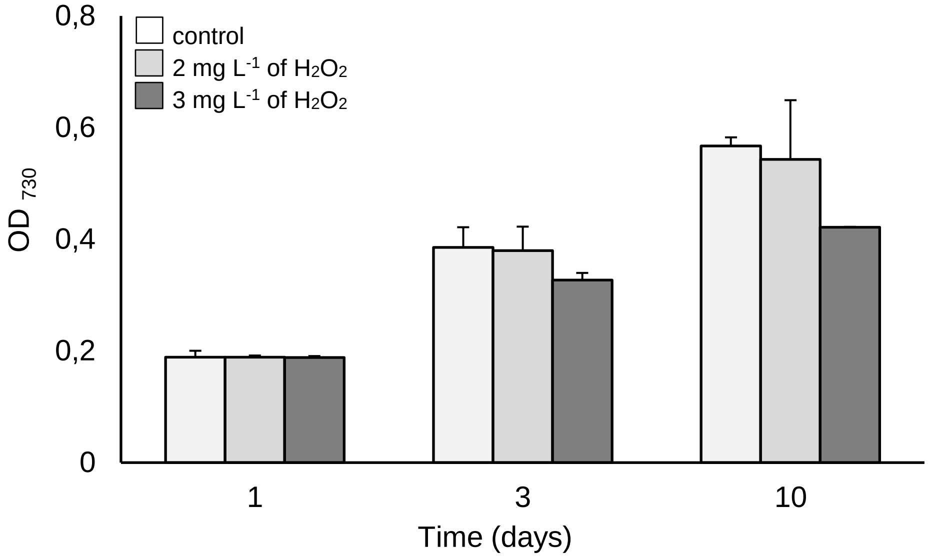

Figure S1 b. The intracellular amount of MC calculated per $OD_{730} = 1$.

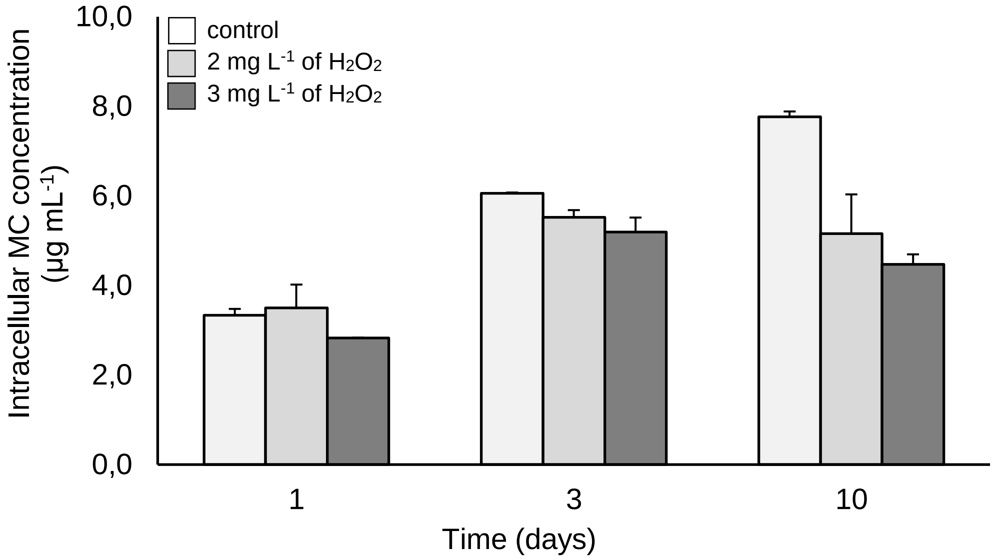

Supplement: Supplementary file 1 [file toxins-11-00725-s001.zip › Figure S1. The growth of H2O2-treated M. aeruginosa culture and the intracellular amount of MC calculated per OD730 = 1.pdf]
