# Supplementary material for: The Effect of a Combined Hydrogen Peroxide-MlrA Treatment on the Phytoplankton Community and Microcystin Concentrations in a Mesocosm Experiment in Lake Ludoš
Source: Toxins (Basel). 2019 Dec 11;11(12):725. doi: 10.3390/toxins11120725 (PMC6950535; doi:10.3390/toxins11120725)
Supplement: Supplementary file 1 [file toxins-11-00725-s001.zip › Table S1. mcyB_M2q and mcyE_M2q primers.pdf]

Table S1. *mcyB*_M2q and *mcyE*_M2q primers

**gene**	**name**	**sequence 5'-3'**	**amplicon length**
mcyB	mcyB_M2qF	CCTCAGACAATCAACGGTTAGT	119
	mcyB_M2qR	AAAGGCAGAAGGCACCATATAA	
mcyE	mcyE_M2qF	CTGGTGGGAAAGGACTGATTTA	95
	mcyE_M2qR	CGCCCTCAAGTCAAGAAAGA	
